# The Combined Administration of Rice Bran and Bacteria Producing Feruloyl Esterase Alleviates DSS‐Induced Ulcerative Colitis in Mice

**DOI:** 10.1002/fsn3.72261

**Published:** 2026-08-21

**Authors:** Le Li, Feiyu Yang, Dan Shen, Jing Sun, Xinyi Pang, Yingjian Lu, Xiangfei Li

**Affiliations:** ^1^ College of Food Science and Engineering Nanjing University of Finance and Economics/Collaborative Innovation Center for Modern Grain Circulation and Safety Nanjing China; ^2^ College of Food Science and Technology Nanjing Agricultural University Nanjing China

**Keywords:** anti‐inflammatory, antioxidant, inflammatory bowel disease, *Lactobacillus johnsonii*, rice bran, ulcerative colitis

## Abstract

Inflammatory bowel disease (IBD) has emerged as a global health concern. This study aimed to evaluate the ameliorative effects of the combined application of rice bran (RB) and *Lactobacillus johnsonii* 23, a producer of feruloyl esterase, on dextran sulfate sodium (DSS)‐induced ulcerative colitis (UC) in mice. High‐yielding ferulic acid esterase bacteria (FAEb) were screened and administered to DSS‐induced UC mice along with rice bran. Disease symptoms, histopathology, oxidative stress, inflammatory factors, and cecal short‐chain fatty acids (SCFAs) were assessed to evaluate the alleviating effects on UC mice. The combined intervention effectively improved body weight changes, disease activity index (DAI) scores, and colon length in UC mice, reversing DSS‐induced epithelial cell necrosis, crypt structural loss, inflammatory cell infiltration, goblet cell depletion, and mucosal erosion and edema. The combination also reduced colonic MDA levels, enhanced SOD, GSH, and GSH‐Px levels, thereby boosting antioxidant capacity. It decreased the expression of proinflammatory cytokines IL‐6, IL‐1β, and TNF‐α while increasing the expression of the anti‐inflammatory factor IL‐10, promoting anti‐inflammatory capacity. Furthermore, the combined application resisted DSS‐induced depletion of SCFAs. The combined administration of rice bran and *L. johnsonii* 23 holds the potential to emerge as a novel dietary supplement for the prevention or treatment of UC.

## Graphical abstract

The synergistic protective effect of rice bran combined with *Lactobacillus johnsonii* 23 on DSS induced ulcerative colitis in mice: evaluated through clinical, biochemical, histopathological, and molecular biology assays.

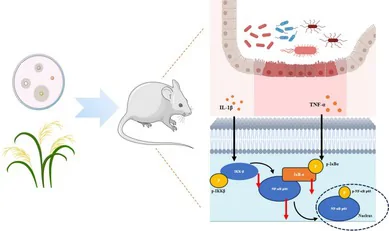

## 1 Introduction

IBD is a gastrointestinal condition resulting from the complex interaction of genetic, gut microbiota, and environmental factors, encompassing Crohn's disease and UC (Caruso et al. 2020; Danne et al. 2023; Verstockt et al. 2023). Currently, it is estimated that approximately 5 million individuals worldwide suffer from UC, and the incidence rate continues to rise, posing a significant burden on global healthcare systems (Ungaro et al. 2017). UC is characterized by high incidence, incurability, and recurrence, manifesting as symptoms such as diarrhea, rectal bleeding, fecal incontinence, and abdominal cramping (Gros and Kaplan 2023). Although the pathogenesis of UC remains incompletely understood, intestinal microbial dysbiosis and barrier impairment have been identified as critical factors promoting UC development (Luo et al. 2023). Microbial dysbiosis leads to the loss of beneficial microbial flora, the increase of pathogenic bacteria and harmful metabolites, and the activation of mucosal immunity, resulting in chronic inflammation (Gilliland et al. 2023; Mills et al. 2022). Conversely, this process stimulates the overgrowth of harmful bacteria and their continuous invasion of the intestinal mucosal and epithelial barriers, triggering intestinal barrier dysfunction (Yue et al. 2024). This impairment accelerates the transfer of luminal antigens and pathogens to the intestinal wall, stimulating the release of proinflammatory cytokines from immune cells in the lamina propria, further exacerbating the inflammatory response (de Souza and Fiocchi 2015).

UC is an incurable, recurrent inflammatory bowel disease with diverse clinical treatment regimens, which can be categorized into chemical drugs, biological agents, small‐molecule targeted medicines, surgical resection and adjuvant nutritional interventions (Argyriou et al. 2026). First‐line conventional drugs include 5‐aminosalicylic acid, glucocorticoids and immunosuppressants: 5‐ASA agents relieve mild UC via local mucosal anti‐oxidation, while glucocorticoids rapidly control acute moderate–severe flares, yet long‐term administration causes severe systemic side effects; immunosuppressants are only suitable for hormone‐dependent patients with risks of myelotoxicity and liver damage (Raine et al. 2021). For refractory moderate–severe UC, anti‐TNF‐α and anti‐IL‐12/IL‐23 monoclonal antibodies as well as oral JAK inhibitors are applied as second‐line targeted therapies, but their high cost, infection risk and drug resistance greatly restrict long‐term application. Total colectomy is reserved for patients with life‐threatening complications, which permanently impairs intestinal physiological function and lowers life quality. Given the limitations of pharmaceutical therapies, natural dietary and probiotic interventions have attracted extensive attention as safe auxiliary strategies for UC prevention and remission maintenance (Verstockt et al. 2023).

RB, a dense source of nutrients and phytochemicals, is rich in various beneficial components such as dietary fiber, polysaccharides, proteins, polyphenols, and vitamins (Pengkumsri et al. 2017). However, its high‐value utilization in humans is limited, with most RB being used as animal feed or fertilizer (Goodyear et al. 2015). This significant waste of RB greatly reduces the added value of agricultural products, necessitating further processing to enhance its utilization. Previous reports indicate that active substances present in RB possess anti‐colitis potential (Pengkumsri et al. 2017; Tanideh et al. 2020). The dietary fiber in RB regulates intestinal ecological balance and serves as a nutrient for specific microbial populations to produce SCFAs (Koh et al. 2016). SCFAs are the primary energy source for intestinal epithelial cells, promoting their proliferation and enhancing intestinal barrier function. Additionally, SCFAs have been shown to promote the maturation and differentiation of colonic regulatory T cells, limiting inflammation driven by innate immune cells (Wu et al. 2023).

RB also contains feruloyl oligosaccharides (FOs), which possess both feruloyl and hydrophilic oligosaccharide groups, conferring dual activity similar to ferulic acid and oligosaccharides. This dual activity enables FOs to exert antioxidant, antibacterial, and immunomodulatory effects (Deng et al. 2022; Yue et al. 2024). However, due to the crosslinking of ester bonds in FOs, they are difficult to utilize in the stomach and small intestine. Instead, they are degraded by enzymes released by the gut microbiota in the large intestine, releasing ferulic acid and oligosaccharides, which then exert their physiological effects (Vitaglione et al. 2008).

Single probiotic supplementation can partially regulate gut microbiota but fails to efficiently release phenolic active substances from grain by‐products. In this study, we innovatively combined RB with feruloyl esterase‐high‐yielding *Lactobacillus johnsonii* 23. The strain‐produced feruloyl esterase hydrolyzes feruloyl oligosaccharides in RB to liberate free ferulic acid and oligosaccharides in the colon. The composite intervention synergistically alleviates DSS‐induced colitis via enhancing intestinal antioxidant capacity, balancing pro−/anti‐inflammatory cytokines, restoring cecal short‐chain fatty acid pools, and repairing damaged colon mucosal barrier. Compared with conventional chemical drugs, this compound dietary supplement is low‐cost, biocompatible, and free of obvious toxic side effects, offering a promising novel adjuvant strategy for UC prevention and auxiliary treatment to compensate for the drawbacks of existing clinical therapeutic options.

## 2 Materials and Methods

### 2.1 Isolation and Screening of FAEb

MRS‐ethyl ferulate (EFA) solid medium was employed for the isolation and screening of FAEb. Briefly, 0.2 g of mouse fecal matter was dissolved in 2 mL of 0.9% NaCl solution, homogenized, and gradiently diluted before plating onto solid MRS‐EFA medium. The plates were incubated at 37°C for 72 h. Single colonies with larger transparent zones were selected and inoculated into MRS liquid medium for cultivation at 37°C. After screening, the strain *L. johnsonii* 23 with the highest FAE‐producing capacity was chosen, mixed with 30% glycerol solution, and stored at −80°C for future use.

### 2.2 16S rRNA Gene Sequencing Identification

16S rRNA gene sequencing was performed according to the method described by Shen (Shen et al. 2022). DNA was extracted from *L. johnsonii* using a bacterial genomic DNA extraction kit (Tiangen, Beijing, China). PCR amplification was conducted using the 16S rRNA universal primers 27 F (5′‐AGAGTTTGATCCTGGCTCAG‐3′) and 1492 R (5′‐GGTTACCTTGTTACGACTT‐3′) with the extracted DNA as the template. The nucleotide sequences of the PCR products were determined by Sangon Biotech Co. Ltd. (Shanghai, China). The sequencing results were then input into the NCBI database (https://www.ncbi.nlm.nih.gov/) for comparison.

### 2.3 Preparation of Test Substances

*L. johnsonii* 23 was inoculated into MRS liquid medium and cultured at 37°C for 18 h. After 2 generations, the bacterial cells were collected by centrifugation at 8000 rpm for 10 min at 4°C, washed twice with sterile saline, and centrifuged again under the same conditions. The bacterial cells were then dissolved in 9% sterile physiological saline and diluted to 5 × 10^9^ CFU/mL for subsequent animal experiments.

### 2.4 Animals and Experimental Design

48 healthy SPF‐grade male C57BL/6J mice aged 6–8 weeks (20 ± 2 g) were purchased from Shanghai SLAC Laboratory Animal Co. Ltd. (SCXK (HU) 2017–0005; Shanghai, China). The animal experimental protocol was approved by the Animal Ethics Committee of the Laboratory Animal Center of Nanjing Agricultural University (permit NO. NJAU. No20210317024) and conducted in accordance with the Regulations for the Administration of Laboratory Animals issued by the State Scientific and Technological Commission of the People's Republic of China. The mice were housed under standard conditions with a temperature of 22°C ± 2°C, relative humidity of 55% ± 5%, and a 12‐h light–dark cycle. They had free access to water and food. A one‐week adaptive feeding period was implemented before the start of the formal experiment.

The dosage of each intervention was determined based on previously reported colitis mouse studies and laboratory pre‐exploration. Briefly, sulfasalazine at 50 mg/kg was selected as the standard positive control dose for DSS‐induced colitis models (Axelsson et al. 1998; Shen et al. 2022). A 20% rice bran‐containing diet was chosen after preliminary feeding trials to balance feeding tolerance and intestinal protective efficacy (Weber et al. 2023). The concentration of *L. johnsonii* 23 suspension was set to 5 × 10^9^ CFU/mL, which was verified to supply sufficient viable bacteria for feruloyl esterase secretion without causing gastrointestinal burden in mice (M. Li et al. 2022; Shen et al. 2022).

**FIGURE 1:**
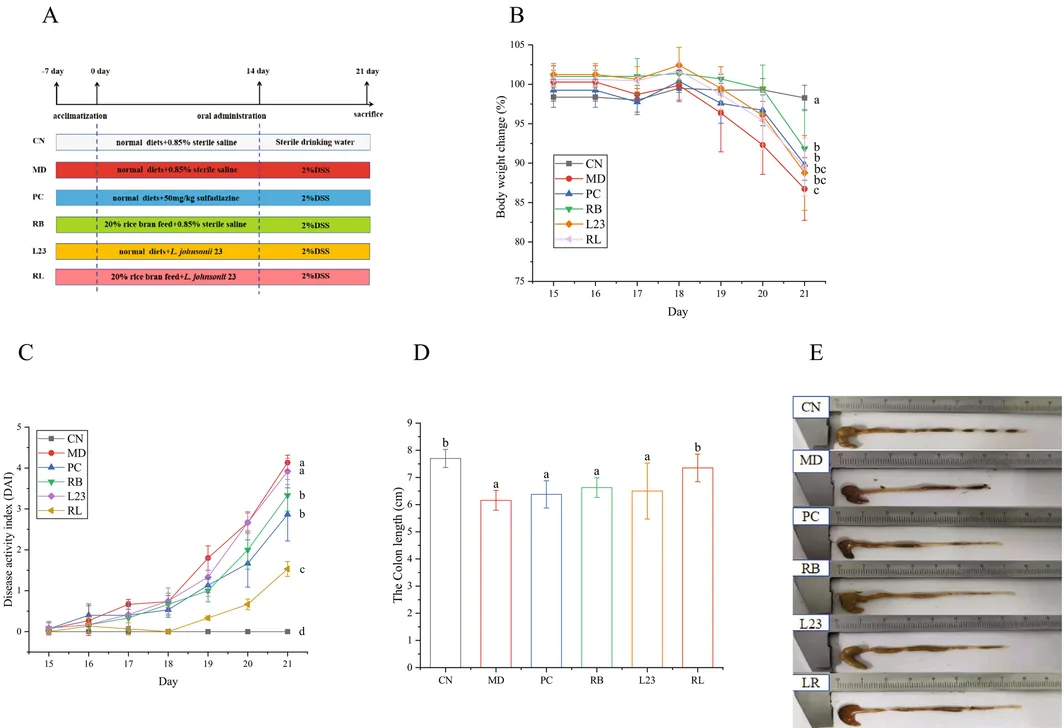
Effects of combined intervention of RB and *L. johnsonii* 23 on UC in mice. (A) Design of animal experimental protocols. (B) Changes in body weight. (C) DAI index. (D) The colon length. (E) Colon length pictures. The data were expressed as mean ± SEM (*n* = 8). Mean values with different superscript letters are significantly different (*p* < 0.05).

The animal experimental procedure is outlined in Figure 1A. 48 mice were randomly divided into seven groups of eight each: control group (CN), DSS group (MD), sulfasalazine group (PC), RB group (RB), *L. johnsonii* 23 group (L23), and RB + *L. johnsonii* 23 group (RL). Mice in the CN and MD groups were orally administered 0.4 mL of 0.85% sterile physiological saline daily. Mice in the PC group received 0.4 mL of sulfasalazine dissolved in 0.85% sterile physiological saline at a dose of 50 mg/kg daily. Mice in the RB group were orally administered 0.4 mL of 0.85% sterile physiological saline daily and fed a diet containing 20% RB. Mice in the L23 group were orally administered 0.4 mL of 5 × 10^9^ CFU/mL *L. johnsonii* 23 suspension daily. Mice in the RL group received 0.4 mL of 5 × 10^9^ CFU/mL *L. johnsonii* 23 suspension daily and were fed a diet containing 20% RB. All groups received sterile drinking water without DSS from days 1 to 13. From days 14 to 21, all groups except the CN group were given a 2% (w/v) DSS (MP Biomedicals LLC) aqueous solution, which was replaced every two days. Body weights of the mice were measured daily during the experiment.

### 2.5 Sample Collection and Processing

Upon completion of the experiment, mice were sacrificed via cervical dislocation after being weighed on the 22nd day. Blood samples were collected and allowed to stand for 15 min before centrifugation at 4°C and 12,000 rpm for 10 min. The supernatant serum was then aspirated for subsequent biochemical analysis. Immediately, the liver, spleen, and kidneys were collected and weighed, and the organ index was calculated using the formula: Organ Index (mg/g) = Organ Weight (mg)/Body Weight (g). The colon was excised, its length measured, and photographed. A 1‐cm colon section was fixed in 4% paraformaldehyde at room temperature and subsequently stained. A 100‐mg colon sample was dissolved in 900 μL of sterile physiological saline, homogenized using a frozen tissue grinder, and centrifuged at 4°C and 12,000 rpm for 10 min. The supernatant was collected for biochemical marker detection. Contents of the cecum were immediately frozen in liquid nitrogen for further analysis.

### 2.6 DAI Scoring

Disease activity was monitored daily from Day 15 to 21 of the experiment. DAI scores were assigned based on body weight loss, stool characteristics, and rectal bleeding using the criteria outlined in Table 1. The DAI was calculated using the formula: (Body Weight Loss Score + Stool Characteristics Score + Rectal Bleeding Score)/3.

**TABLE 1:** Effects of combined administration of RB and *L. johnsonii* 23 on organ indexes.

Indicators	CN	MD	PC	RB	L23	RL
Liver (mg/g)	38.38 ± 2.77^a^	40.35 ± 1.69^a^	39.37 ± 2.48^a^	39.64 ± 2.83^a^	40.25 ± 4.84^a^	39.49 ± 3.33^a^
Spleen (mg/g)	2.56 ± 0.50^a^	5.04 ± 1.36^c^	3.45 ± 0.37^b^	2.87 ± 0.43^ab^	3.54 ± 0.62^b^	3.47 ± 0.68^b^
Kidney (mg/g)	12.9 ± 0.81^a^	12.37 ± 0.77^a^	12.32 ± 0.66^a^	12.3 ± 1.12^a^	12.59 ± 1.20^a^	12.49 ± 0.55^a^

*Note:* Data are expressed as mean ± SEM (*n* = 8); values in the same column with different superscript letters are significantly different (*p* < 0.05).

### 2.7 Biochemical Indices Determination

The concentrations of malondialdehyde (MDA), superoxide dismutase (SOD), glutathione (GSH), and glutathione peroxidase (GSH‐PX) in colon homogenates were measured using enzyme‐linked immunosorbent assay (ELISA) kits obtained from Jiancheng Bioengineering Institute (Nanjing, Jiangsu, China). Serum concentrations of interleukin‐1β (IL‐1β), IL‐6, IL‐10, and tumor necrosis factor‐α (TNF‐α) were determined using ELISA kits.

### 2.8 Histological Analysis

Colon tissues were fixed in 4% paraformaldehyde for 24 h, rinsed with ultrapure water to remove the fixative, dehydrated in a graded series of ethanol solutions, and cleared in xylene. The colon tissue sections were embedded in paraffin, cut into 4‐μm slices, and stained with hematoxylin and eosin (HE) as well as Alcian blue and Periodic acid‐Schiff (AB‐PAS). For AB‐PAS staining, colon sections were dewaxed and washed, stained with Alcian blue for 10 min and washed, stained with periodic acid for 5 min and washed, stained with Schiff's reagent for 10 min and washed, dehydrated in ethanol, cleared in xylene, and sealed with neutral glue. All sections were observed under an Olympus microscope (Tokyo, Japan).

### 2.9 Determination of SCFAs in Cecal Contents

Cecal contents were collected into 2‐mL sterile centrifuge tubes. A 500‐μL aliquot of saturated NaCl solution was added to dissolve and homogenize the contents. Then, 20 μL of 10% sulfuric acid was added for acidification, and the mixture was vortexed for 30 s. Subsequently, 800 μL of ether was added, and the mixture was vortexed for another 30 s. The solution was centrifuged at 4°C and 14,000 rpm for 15 min, and the supernatant was collected into a 2 mL centrifuge tube containing 0.25 g of anhydrous Na_2_SO_4_ (drying agent) for incubation for 10 min. After centrifugation under the same conditions, the supernatant was collected, filtered through a 0.22‐μm organic membrane, and analyzed using gas chromatography–mass spectrometry (GC–MS). GC–MS conditions were as follows: Rtx‐Wax column (30 m × 0.25 mm × 0.25 μm); helium carrier gas flow rate of 2 mL/min; injection volume of 1 μL; split ratio of 10:1; ion source temperature of 220°C; and inlet temperature of 240°C. The temperature program used was as follows: initial temperature of 100°C; increased to 140°C at a rate of 7.5°C/min; increased to 200°C at a rate of 60°C/min; and maintained for 3 min. The concentrations of each SCFA (μmol/g) were calculated using the external standard method in full scan mode.

### 2.10 Statistical Analysis

All data are expressed as mean ± standard error of the mean (SEM). The one‐way analysis of variance (ANOVA) was used to analyze all data, followed by Duncan's multiple range test. A *p*‐value < 0.05 was considered statistically significant. Graphical presentations were performed using Origin 9.0 software (OriginLab Corporation, Northampton, MA), and all statistical analyses were conducted using SPSS 16.0 software (SPSS, Chicago, IL).

## 3 Results

### 3.1 Screening and Identification of FAEb

MRS‐ethyl ferulate (EFA) opaque solid medium was adopted to screen FAE‐producing strains. Briefly, mouse fecal samples were gradient diluted and spread on MRS‐EFA plates, followed by 72 h anaerobic incubation at 37°C. Strains with distinct transparent hydrolysis zones around colonies were preliminarily selected, as the clear circle diameter directly reflected extracellular feruloyl esterase activity. All candidate single colonies were inoculated into MRS liquid medium for subculture, and the fermentation supernatant of each strain was further verified by the transparent circle method to exclude false‐positive strains. The isolate with the largest transparent zone in both colony and supernatant re‐screening was identified via 16S rRNA gene sequencing. Sequence alignment against the NCBI database confirmed the strain belonged to *Lactobacillus johnsonii* , which was designated as *L. johnsonii* 23 for subsequent animal experiments.

### 3.2 Synergistic Effect of RB and FAE‐Producing Bacteria on Disease Symptoms in Colitis Mice

A colitis model was established in mice by inducing with 2% DSS to analyze the synergistic alleviating effects of RB and FAE‐producing bacteria on colitis mice. As shown in Figure 1B, compared with the MD group, the RB, L23, and RL groups all significantly alleviated the decrease in body weight in mice. The DAI of mice in the RB, L23, and RL groups was significantly reduced (*p* < 0.05), with the most significant decrease observed in the RJ group (Figure 1C). Shortening of colon length is a critical pathological event in DSS‐induced colitis. As can be seen from Figure 1D, compared with the MD group, the colon lengths of the RB, L23, and RL treatment groups were significantly increased (*p* < 0.05), with a more pronounced increase in the RL group.

### 3.3 Synergistic Effect of RB and FAE‐Producing Bacteria on Organ Indices in Colitis Mice

As shown in Table 1, DSS‐induced colitis led to spleen enlargement in mice. Compared with the MD group, the RB, L23, and RL groups all significantly reduced the spleen index of mice (*p* < 0.05). However, there were no significant differences in liver and kidney indices between the treatment groups and the control group.

### 3.4 Synergistic Effect of RB and FAE‐Producing Bacteria on Histopathology in Colitis Mice

**FIGURE 2:**
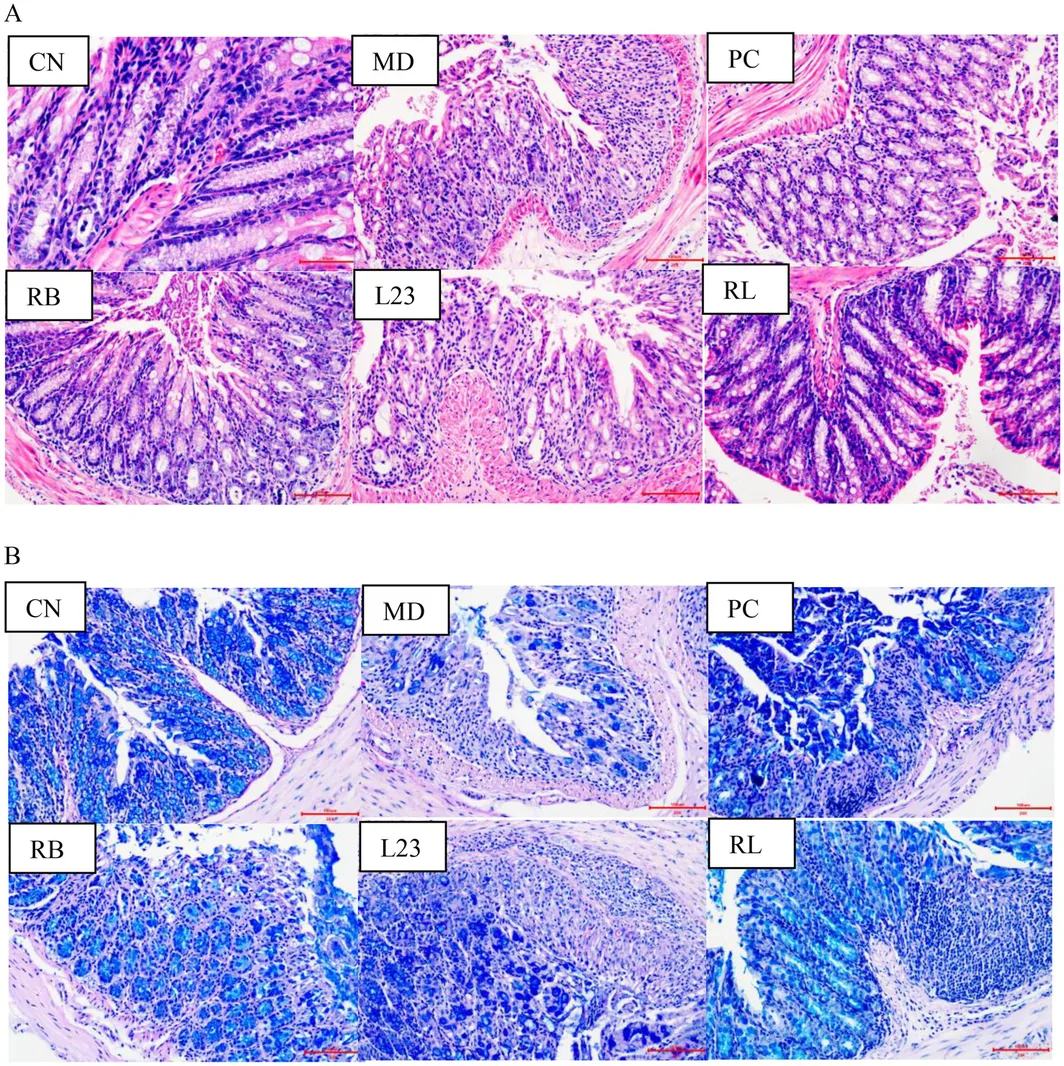
Effect of combined intervention of RB and *L. johnsonii* 23 on colonic histopathology in mice. (A) Colon HE staining. (B) Colonic AB‐PAS staining. Images of colon tissue stained by HE and AB‐PAS were observed at 200× magnification.

H&E‐stained colon histological analysis clearly revealed severe damage to the colon of mice with DSS‐induced colitis, characterized by necrosis of epithelial cells, loss of crypt structure, inflammatory cell infiltration, decreased goblet cells, and mucosal erosion and edema (Figure 2A). After treatment, colon tissue recovery was observed in the RB and L23 groups, with reduced epithelial cell necrosis, normal crypt structure, fewer inflammatory cell infiltrates, and increased goblet cell numbers. The RL group exhibited even better improvement, with only minor structural abnormalities in the intestinal tissue, limited epithelial cell necrosis and crypt structure damage, and almost undetectable inflammatory infiltration.

Figure 2B shows that the mucus layer of colon tissue in the MD group was severely damaged, and goblet cells and mucin were significantly reduced. The number of goblet cells containing acidic mucopolysaccharides was restored in the RL group. The results indicate that the combined application of rice bran and *L. johnsonii* 23 can prevent tissue damage induced by DSS by increasing goblet cells in colon tissue and improving the mucus layer.

### 3.5 Synergistic Effect of RB and FAE‐Producing Bacteria on Oxidative Stress in Colitis Mice

**FIGURE 3:**
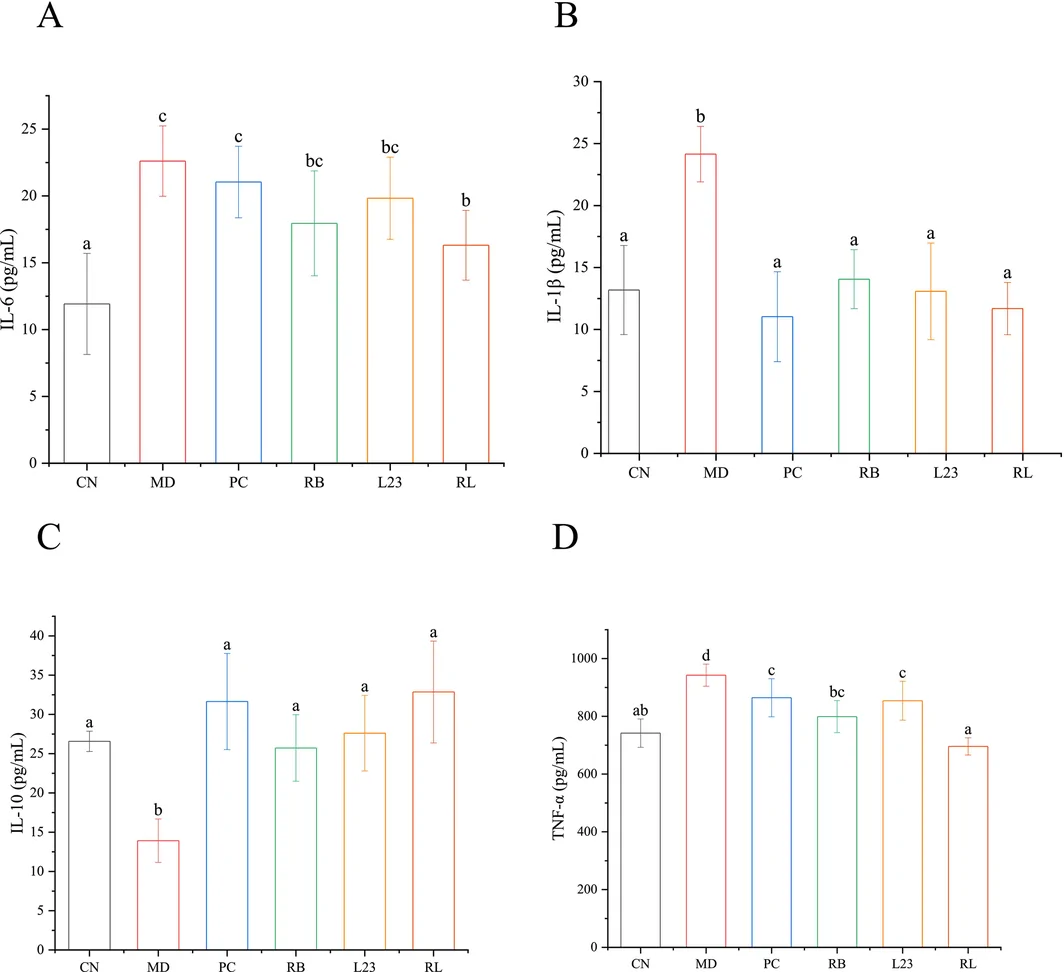
Effects of combined intervention of RB and *L. johnsonii* 23 on inflammatory factors. (A) IL‐6. (B) IL‐1β. (C) IL‐10. (D) TNF‐α. The data were expressed as mean ± SEM (*n* = 8). Mean values with different superscript letters are significantly different (*p* < 0.05).

The oxidative damage in the colon of mice is shown in Figure 3. Compared with CN, the level of MDA in the MD group was significantly elevated (*p* < 0.05), while the levels of SOD, GSH, and GSH‐Px were significantly reduced (*p* < 0.05), indicating that DSS induction led to oxidative stress and disrupted redox homeostasis in the body. After intervention, compared with the MD group, the RB, L23, and RL treatment groups all significantly reduced the level of MDA and significantly increased the levels of SOD, GSH, and GSH‐Px (*p* < 0.05). Furthermore, compared with the RB and L23 groups, the RL group exhibited stronger resistance to oxidative stress.

### 3.6 The Collaborative Effect of RB and Feruloyl Esterase‐Producing Bacteria on Inflammatory Factors in Mice With Colitis

**FIGURE 4:**
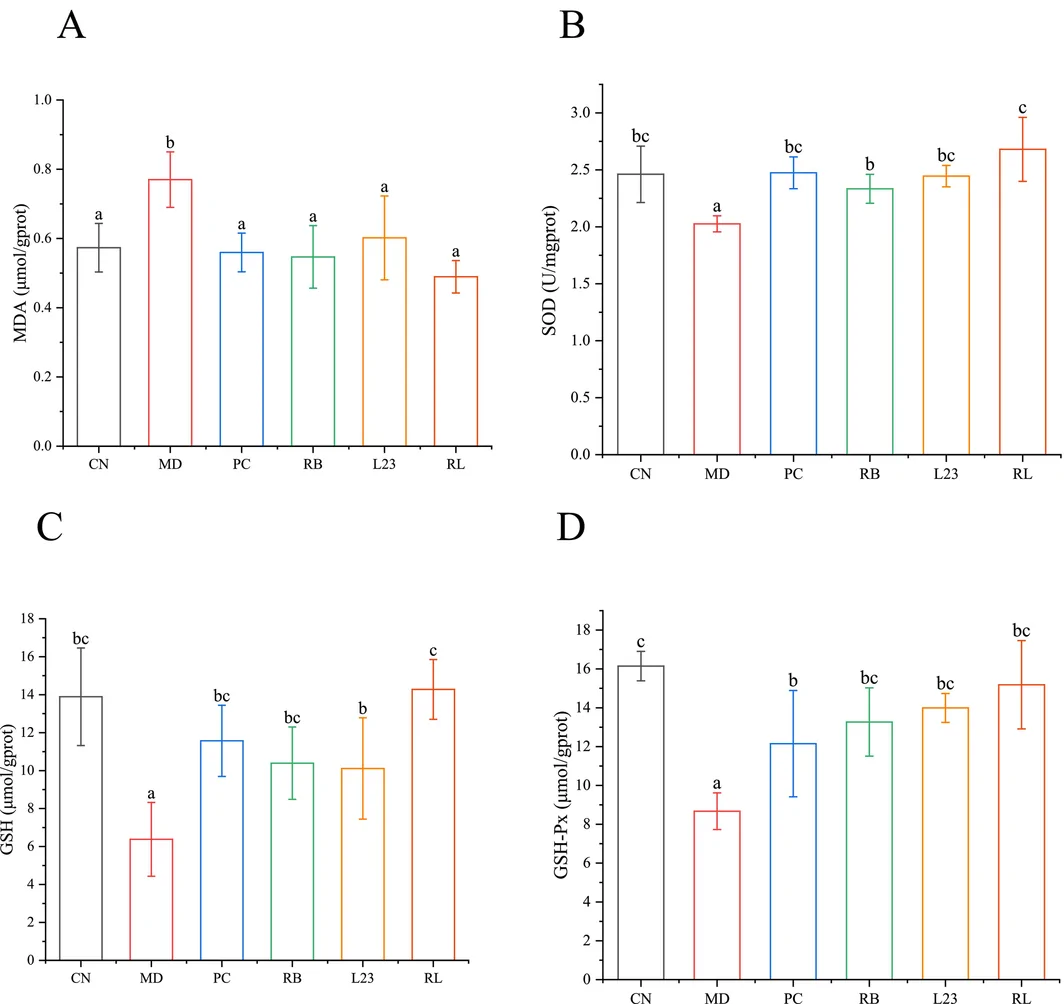
Effects of combined intervention of RB and *L. johnsonii* 23 on oxidative stress parameters. (A) MDA. (B) SOD. (C) GSH. (D) GSH‐Px. The data were expressed as mean ± SEM (*n* = 8). Mean values with different superscript letters are significantly different (*p* < 0.05).

As shown in the Figure 4, the levels of inflammatory factors in the serum of mice were measured. Compared with the CN group, DSS induction led to a significant increase (*p* < 0.05) in the expression of proinflammatory factors IL‐6, IL‐1β, and TNF‐α, and a significant decrease (*p* < 0.05) in the expression of the anti‐inflammatory factor IL‐10, indicating inflammatory damage to the colon. After intervention, compared with the MD group, the RB, L23, and RL treatment groups all significantly reduced the levels of IL‐6, IL‐1β, and TNF‐α (*p* < 0.05) and increased the level of the anti‐inflammatory factor IL‐10 (*p* < 0.05). Moreover, the RL group exhibited stronger anti‐inflammatory ability compared to the RB and L23 groups.

### 3.7 The Collaborative Effect of RB and Feruloyl Esterase‐Producing Bacteria on SCFAs in the Cecal Contents of Mice With Colitis

**FIGURE 5:**
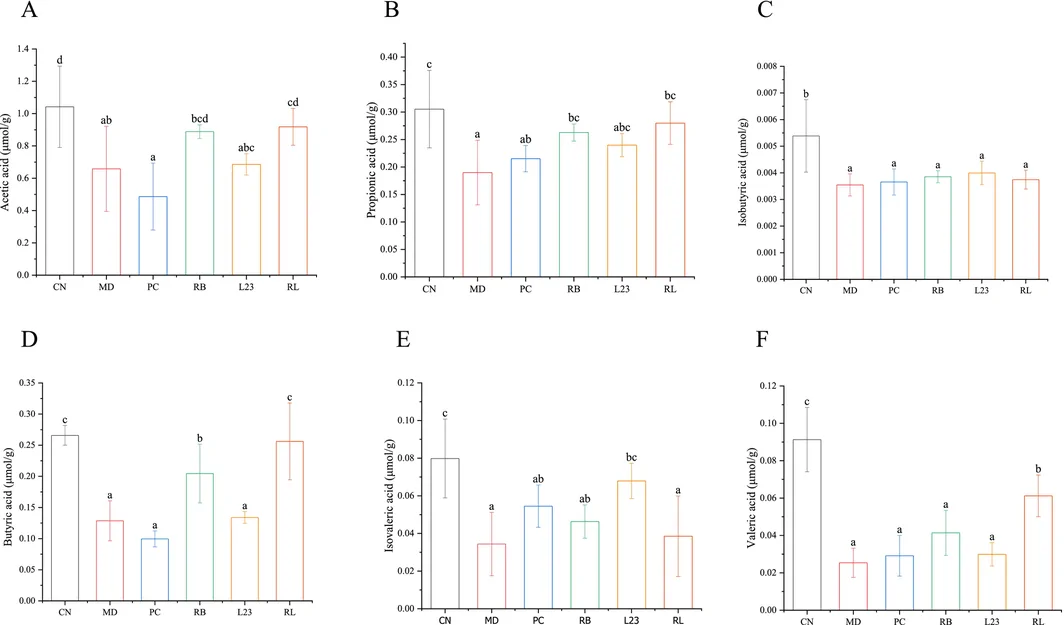
Effects of the combined intervention of RB and *L. johnsonii* 23 on SCFA: (A) Acetic acid; (B) Propionic acid; (C) Isobutyric acid; (D) Isobutyric acid; (E) Isovaleric acid; (F) Valeric acid. The data were expressed as mean ± SEM (*n* = 8). Mean values with different superscript letters are significantly different (*p* < 0.05).

Changes in the six SCFAs are depicted in the Figure 5. Compared with the CN group, DSS induction resulted in a consumption of acetic acid, propionic acid, isobutyric acid, butyric acid, isovaleric acid, and valeric acid (*p* < 0.05). After intervention, compared with the MD group, the content of acetic acid, propionic acid, and butyric acid significantly increased in the RB group (*p* < 0.05), while the content of acetic acid and isovaleric acid significantly increased in the L23 group (*p* < 0.05). Additionally, the content of acetic acid, propionic acid, butyric acid, and valeric acid significantly increased in the RL group (*p* < 0.05). These results indicate that the collaboration of RB and feruloyl esterase‐producing bacteria can effectively counter the consumption of SCFAs in mice with colitis.

## 4 Discussion

UC, an immune‐mediated intestinal inflammatory disease, has rapidly increasing incidence rates and has become a significant global public health concern. Multiple lines of evidence suggest that gut microbiota dysbiosis and the disruption of the intestinal mucosal barrier contribute to the occurrence and progression of UC (Cao et al. 2023; Gu et al. 2021). This study explored the collaborative effect of RB and the feruloyl esterase‐producing bacterium *L. johnsonii* 23 on DSS‐induced colitis in mice. We provide evidence that the simultaneous supplementation of RB and *L. johnsonii* 23 effectively ameliorates DSS‐induced colitis. This may be due to their synergistic action in releasing active substances from RB, exerting anti‐inflammatory and antioxidant effects, and improving colitis in mice.

Dietary and functional foods contain numerous functional active factors that serve as effective alternatives in the prevention and management of IBD (M. Li et al. 2022). RB is rich in various nutrients and possesses excellent health‐promoting functions and biological activities. In particular, ferulic acid possesses antioxidant and anti‐inflammatory properties. However, most ferulic acid in grains is bound to the cell wall through ester bonds, rendering it inaccessible to enzymatic degradation in the human digestive tract. Recent studies have shown that the fermentation of RB using microbial fungi or bacteria can produce extremely diverse metabolites and enhance its biological activity (Islam et al. 2021; Rusbana et al. 2020). Therefore, we hypothesized that the collaborative action of *L. johnsonii* 23 and RB could release active substances such as ferulic acid and oligosaccharides to alleviate intestinal inflammation, and we attempted to use *L. johnsonii* 23 and RB to mitigate colitis.

Body weight changes, DAI scores, colon length, and colon histopathological scores are crucial markers for evaluating the severity of UC (L. Li et al. 2023). DSS induction disrupts the systematic intestinal tissue structure, intestinal mucus barrier, and tight junctions between intestinal cells, leading to inflammatory cell infiltration (Gancarcikova et al. 2020). In this study, both individual and combined administration of RB and *L. johnsonii* 23 improved these UC markers. As expected, the combined application of RB and feruloyl esterase‐producing bacteria exhibited a superior effect in ameliorating UC.

Among immune regulatory factors, oxidative stress is considered a major mechanism promoting IBD development (Hwang et al. 2020). While moderate levels of reactive oxygen species (ROS) serve as second messengers regulating intracellular homeostasis and redox signaling, among other biological functions, to promote cell proliferation and tissue renewal, excessive ROS levels interact with DNA, proteins, and lipids, disrupting various metabolic and cellular homeostases, leading to cellular damage (Zuo et al. 2022). Furthermore, excessive ROS triggers abnormal activation of the intestinal immune system, weakens the mucus barrier, disrupts tight junctions, and reduces antimicrobial peptide secretion, further exacerbating UC progression (M. Li et al. 2022). Here, we found that individual administration of RB and *L. johnsonii* 23 increased the activity of key antioxidant enzymes in the colon of DSS‐induced mice. The RB group had a higher impact on SOD activity in UC mice, while the feruloyl esterase‐producing bacteria group showed a greater influence on CAT activity. The combined administration of RB and *L. johnsonii* 23 had the most significant impact on antioxidant enzyme activity, possibly due to the synergistic action between the two, leading to the release of free ferulic acid and oligosaccharides.

In UC, the persistent accumulation of inflammatory cells in the intestine is a crucial mechanism leading to inflammatory damage (Lee et al. 2018). Recognition of PAMPs associated with the intestinal microbiota and DAMPs related to oxidative stress activates the immune system to release a series of inflammatory mediators, attracting macrophages, neutrophils, and T lymphocytes into the intestine (Gong et al. 2019; Liston and Masters 2017). Subsequently, these inflammatory cells release inflammatory cytokines and ROS, leading to sustained damage to the intestinal mucosa and the expansion of the inflammatory response (Gilliland et al. 2023). In this study, DSS induction increased the levels of proinflammatory cytokines IL‐6, IL‐1β, and TNF‐α in the serum of mice, while anti‐inflammatory cytokine IL‐10 decreased. Individual intervention with RB and feruloyl esterase‐producing bacteria reversed this trend to varying degrees, with combined administration exhibiting a better anti‐inflammatory effect. Additionally, proinflammatory cytokines exacerbate intestinal inflammation by stimulating the activation of the NF‐κB signaling pathway (Shen et al. 2022). Signaling through this pathway is transmitted to IKKβ, leading to the phosphorylation of IκBα and the release of NF‐κB p65 into the nucleus, initiating or enhancing the transcription of genes related to inflammatory responses and apoptosis, ultimately promoting intestinal inflammation (Capece et al. 2022). Similarly, the combined administration of RB and *L. johnsonii* 23 showed stronger anti‐inflammatory capabilities. This may be partially attributed to the ability of feruloyl esterase to release free ferulic acid and oligosaccharides, exerting anti‐inflammatory and antioxidant effects.

SCFAs are a group of metabolites mainly produced by the fermentation of dietary fiber by intestinal microbiota. SCFAs play crucial roles in promoting immune cell function, energy metabolism, and intestinal homeostasis (Chen et al. 2023). Additionally, increased SCFAs levels in the cecum can lower the pH, inhibiting the growth and colonization of harmful bacteria (Guo et al. 2023). However, chronic UC leads to depletion of SCFAs in the cecal contents (Parada Venegas et al. 2019). In this study, compared to DSS‐induced model mice, combined intervention with RB and *L. johnsonii* 23 increased the levels of acetic acid, isobutyric acid, and butyric acid in the cecal contents of UC mice, with a superior effect compared to sulfasalazine treatment. This may be attributed to the utilization of a large amount of dietary fiber in RB by the intestinal microbiota for metabolism and SCFAs production. Additionally, *L. johnsonii* 23 produces feruloyl esterase that can break down dietary fiber, improving its utilization and increasing the production of SCFAs in the body. Notably, butyric acid, as an important SCFA, provides energy for colon cells and enhances the mucus barrier (Mohamed Elfadil et al. 2023). Therefore, these results suggest that the alleviation of UC by combined intervention with RB and *L. johnsonii* 23 is partially explained by modulation of the intestinal microbiota and its metabolites.

Compared with mainstream clinical UC treatments, the combined intervention of rice bran and *L. johnsonii* 23 shows distinct advantages and clear application positioning. Conventional chemical drugs (5‐ASA, glucocorticoids, immunosuppressants), biological monoclonal antibodies, and oral JAK inhibitors effectively relieve intestinal inflammation but are accompanied by obvious drawbacks including various systemic side effects, high treatment costs, infection risks, and drug resistance (Verstockt et al. 2023), while total colectomy permanently damages intestinal physiological function and reduces patients' quality of life (L. Li et al. 2023; Lu et al. 2026). As a natural synbiotic formula derived from agricultural by‐products and probiotics, this combination exerts synergistic antioxidant, anti‐inflammatory, and gut microbiota‐regulating effects via releasing free ferulic acid and elevating cecal SCFAs, with no detectable organ toxicity in mice and much lower economic cost. Nevertheless, it cannot replace pharmaceutical agents for severe acute UC flares; its core value lies in serving as a safe auxiliary dietary strategy to prevent UC onset and maintain remission for mild‐to‐moderate patients, which outperforms single rice bran or single strain supplementation due to the feruloyl esterase‐mediated synergistic activation of rice bran bioactive substances.

## Author Contributions

**Yingjian Lu:** funding acquisition, methodology, data curation. **Xiangfei Li:** writing – original draft, investigation, writing – review and editing. **Xinyi Pang:** resources, project administration. **Feiyu Yang:** methodology, conceptualization, software. **Dan Shen:** methodology, validation, formal analysis. **Le Li:** software, data curation. **Jing Sun:** project administration, visualization.

## Funding

This work was supported by the National Natural Science Foundation of China (32572590), the Priority Academic Program Development of Jiangsu Higher Education Institutions (PAPD), the Cross‐Innovation Open Project of Food Flavor and Health, Beijing Technology & Business University.

## Conflicts of Interest

The authors declare no conflicts of interest.

## Data Availability

The datasets generated and analyzed during the current study are not publicly accessible, but can be obtained from the corresponding author upon reasonable request.
